# Supplementary material for: Protocol for a randomised, double-blind trial of a chronotherapeutic mobile health (mHealth) behaviour change intervention to optimise light exposure among older adults aged ≥ 60 years in Singapore (LightSPAN)
Source: BMC Geriatr. 2026 Feb 25;26:446. doi: 10.1186/s12877-026-07105-6 (PMC13040864; doi:10.1186/s12877-026-07105-6)
Supplement: Supplementary file 1 — Supplementary Material 1. [file 12877_2026_7105_MOESM1_ESM.pdf]

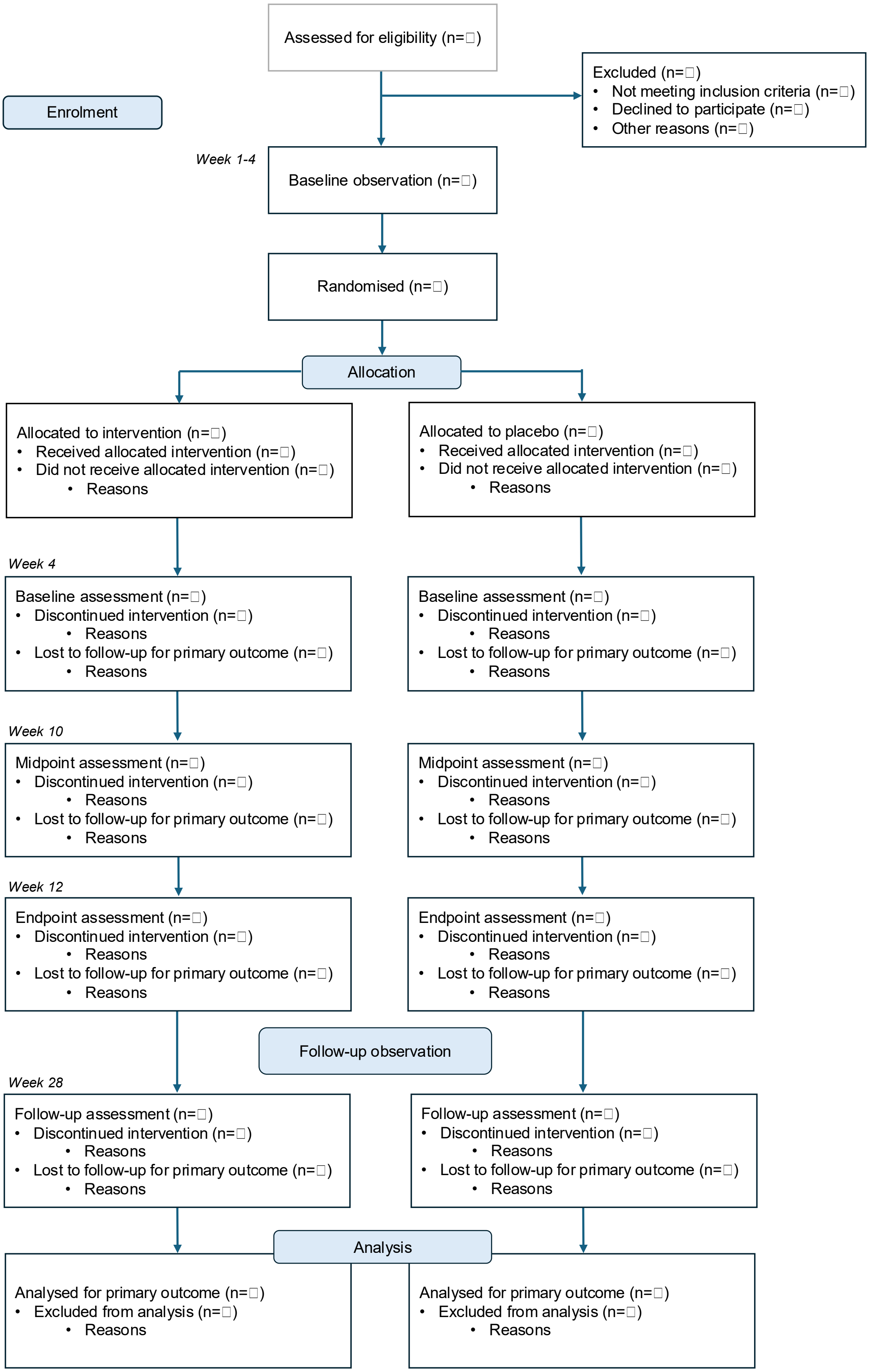

Supplementary Figure 1. CONSORT flow diagram illustrating enrolment, randomisation, allocation, assessment time points, follow-up, and analysis for the LightSPAN study protocol.
